# An Ensemble Learning Based Framework for Traditional Chinese Medicine Data Analysis with ICD-10 Labels

**DOI:** 10.1155/2015/507925

**Published:** 2015-10-01

**Authors:** Gang Zhang, Yonghui Huang, Ling Zhong, Shanxing Ou, Yi Zhang, Ziping Li

**Affiliations:** ^1^School of Automation, Guangdong University of Technology, Guangzhou 510006, China; ^2^Department of Radiology, Guangzhou General Hospital of Guangzhou Military Command, Guangzhou 510010, China; ^3^Department of Plastic and Reconstructive Surgery, The First Affiliated Hospital of Sun Yat-Sen University, Guangzhou 510080, China; ^4^The Second Affiliated Hospital of Guangzhou University of Chinese Medicine, Guangzhou 510120, China

## Abstract

*Objective.* This study aims to establish a model to analyze clinical experience of TCM veteran doctors. We propose an ensemble learning based framework to analyze clinical records with ICD-10 labels information for effective diagnosis and acupoints recommendation. *Methods.* We propose an ensemble learning framework for the analysis task. A set of base learners composed of decision tree (DT) and support vector machine (SVM) are trained by bootstrapping the training dataset. The base learners are sorted by accuracy and diversity through nondominated sort (NDS) algorithm and combined through a deep ensemble learning strategy. *Results.* We evaluate the proposed method with comparison to two currently successful methods on a clinical diagnosis dataset with manually labeled ICD-10 information. ICD-10 label annotation and acupoints recommendation are evaluated for three methods. The proposed method achieves an accuracy rate of 88.2%  ±  2.8% measured by zero-one loss for the first evaluation session and 79.6%  ±  3.6% measured by Hamming loss, which are superior to the other two methods. *Conclusion.* The proposed ensemble model can effectively model the implied knowledge and experience in historic clinical data records. The computational cost of training a set of base learners is relatively low.

## 1. Introduction

In the study of Traditional Chinese Medicine (TCM), clinical experience of veteran doctors plays an important role in both theoretical research and clinical research [1]. The clinical experience is often recorded in a semistructural or unstructured manner, since most of them have a relatively long history. Some of them are manually organized in simple categories or even in plain text. In data mining and machine learning applications, structural inputs are required for computational models [2]. However, there is valuable knowledge in these clinical experience records; for example, they can be used for classification or association rule mining to find patterns of disease diagnosis and Chinese medical ZHENG diagnosis, or for identification of core elements of ZHENG, the relation between herbal medicine formula and different ZHENG and disease, and the common law of clinical diagnosis [3, 4].

There are at least three challenges in building the computational model for analysis clinical records of veteran TCM doctors. The first is that the target data record set for analysis is multimodal with many correlated factors, which means that the data samples are not generated from a single model, but several unknown models or their combination. Hence a simple parameter model cannot capture the generative laws of such data [5, 6]. The second is that the prior knowledge from TCM theory and clinical treatment is available, and they are totally informally organized and even ambiguous, which cannot be directly used in building analysis models. The third is that the data is unstructured, which means that effective feature representations are often unavailable [7].

Currently there some studies on TCM data analysis with machine learning models. We briefly review some work closely related to this work. Di et al. [8] proposed a clinical outcome evaluation model based on local learning for the efficacy of acupuncture neck pain caused by cervical spondylosis. They introduced a local learning method, by defining a distance function between treatment records of each patient. When evaluating the efficacy of acupuncture for a patient, the model selects *p* samples most close to the test sample. The model significantly reduces the computational cost when the dataset is large. However, their model requires a structural input and cannot process data stored in plain text. Liang et al. [9] proposed a multiview KNN method for subjective data of TCM acupuncture treatment to evaluate the therapeutic effect of neck pain. They regard the clinical records as data samples with multiple view, each of which refers to a subset of attributes. And different views are disjointed from each other. The model fully makes use of information from different views. A boosting-style method is used to combine models associated with different views together. Zhang et al. [10, 11] proposed a kernel decision tree method for TCM data analysis. Their model processes data in a feature space induced by a kernel function, which is effective for the multimodal data. However, the prior knowledge cannot be explicitly expressed in the feature space, which limits its further application.

To tackle the aforementioned challenges, in this paper, we propose to adopt the recently proposed deep ensemble learning method to build our analysis model. Deep ensemble learning is an extension of ensemble learning, which is a famous topic in machine learning research [12–14]. Ensemble learning makes a weighted combination of a set of base learners to form a combined learner as the final model. Equation (1) shows the general form of ensemble of base learners:

(1) $$\begin{array}{c} h_{ens}\left(\;x\;\right)=\overset{m}{\underset{i=1}{\sum }}w_{i}\cdot h_{i}\left(\;x\;\right), \end{array}$$

where *h*_*i*_ is a set of base learners of at least some difference and *w* is a weight vector with constraints ∑_*i*_ *w*_*i*_ = 1, *w*_*i*_ ≥ 0. To avoid the overfitting problem of the ensemble learner *h*_ens_, a regularization prior should be imposed on *w* [15]. A common regularization prior is the sparsity of *w*, meaning that more 0 in *w* is preferable. Or one can impose a normal distribution on *w*.

The quality of the set of base learners and *w* fully controls the performance of the ensemble learner [16]. There are three methods to determine the best ensemble of a set of base learners [17]. The first is the selective ensemble, which selects small parts of base learners by some criteria and combines them using a majority voting strategy. This kind of method in fact imposes a prior on *w* that only a small number of elements in *w* can be nonzero, as well as the equal weight for each remaining learner. The second method finds the optimal *w* through solving an optimization as follows:

(2) $$\begin{array}{c} \underset{w}{\min}\;\text{Loss}\left(\;\underset{i}{\sum }w_{i}\cdot h_{i},D\;\right)+\Omega \left(\;w\;\right). \end{array}$$

This kind of method finds the optimal *w* such that the ensemble achieves the minimal loss and the best regularization on the evaluation set parameterized by *w*. Since the optimization problem is not convex for most loss evaluation functions, it may not be solved analytically. The third method is an iterative method that initializes the weights randomly and adjusts them through a iterative procedure. The famous Adaboost algorithm falls into this kind [18]. The Adaboost algorithm adopts very simple principle when finding the optimal weights; that is, if a candidate base learner has a good performance on the training dataset and is different from others, its weight can be increased by the algorithm. The idea of Adaboost is to find a subset of base learners of high quality whose diversity is also high [19].

However, the above three methods do not fully meet the requirement of the problem of TCM data analysis. The concept class implied in our dataset is of complex structure, or in another word, its VC dimension is extremely large, leading to a complex class boundary. Simple or shallow function classes may suffer from lack of representation capability. Motivated by the current research process of ensemble learning and deep learning, we propose to use deep ensemble learning for our analysis task. Different from classical ensemble learning, deep ensemble learning tries to tackle the problem of multimodal analysis and extends the bound of generalization ability of the ensemble learner. A key advantage of deep ensemble learning is that deep models can be used as base learners, which extends the representation capability to a great extent [20, 21].

**Figure 1:**
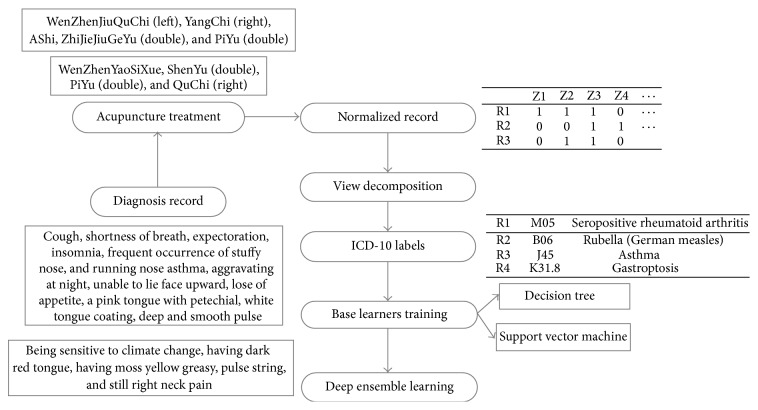
The main idea of this paper.

Figure 1 shows the main idea of this paper, as well as an example of the clinical data to be analyzed.

Deep ensemble learning method adopts a capacity-conscious criterion to evaluate the quality of base learners. Different from the famous accuracy-diversity selective ensemble framework, the deep ensemble learning methods try to directly minimize the error bound according to the current training dataset.

The remainder of this paper is organized as follows. In Section 2 we present the main methods, including the self-adaptive region cutting method, stacked autoencoder training algorithm, and MIML model. In Section 3 we present the settings of evaluation of the proposed method and report the evaluation results on a real clinical dataset at different multiple-label classification criteria. And finally we conclude the paper in Section 4.

## 2. Deep Ensemble Learning

### 2.1. Problem Definition

Before going further, we formally define the problem to be solved. Let *D* = {(*x*_1_, *y*_1_, *z*_1_), (*x*_2_, *y*_2_, *z*_2_),…, (*x*_*n*_, *y*_*n*_, *z*_*n*_)} be a set of TCM clinical records and the corresponding ICD-10 labels, where *x*_*i*_ ∈ *X*⊆0, 1^*d*^ is the representation of each data sample in *D*. Each element of *x*_*i*_ is denoted as *x*_*ij*_, indicating whether an acupoint or ZHENG is included in the treatment plan. The acupoint and ZHENG information are extracted through a simple key word matching procedure. *y*_*i*_ ∈ *L* is an ICD-10 label associated with the *i*th record. *z*_*i*_ ∈ *Z* is the TCM diagnosis of the *i*th clinical sample. When an acupoint or ZHENG is found, the correspond element in *x*_*i*_ is set to 1, and 0 otherwise. The goal is to find a function *h* : *X* → *L* × *Z* that achieves minimal loss on a training dataset *D*_train_, given a predefined loss function, for example, zero-one loss.

### 2.2. Selective Ensemble and Learners Sorting

Selective ensemble is an ensemble strategy that sorts the base learners with some criteria and then selects the learners at the top of the list to ensemble. Three criteria are used in this study. The first is accuracy, which evaluates how the model output matches the ground truth label [22]. Since the output of *h* is a pair of labels, that is, *h*(*x*_*i*_) = (*y*_*i*_, *z*_*i*_), the simple zero-one loss is not suitable in this case. We define a new accuracy as follows:

(3) AcchD=1D∑i=1Dα·δhxiy,yi+β·δhxiz,zi+γ·Δhxi,yi,zi,

where *α*, *β*, and *γ* are parameters controlling the importance of TCM diagnosis, ICD-10, and both. The indicator function *δ*(*x*, *y*) = 1 if *x* = *y* and 0 otherwise. Δ is also an indicator function that evaluates two tuples.

The second criterion is diversity which evaluates the difference between base learners. According to the theory of ensemble learning, an ensemble of learners that are different from each other may achieve better performance. There are some diversity definitions proposed in the literature of ensemble learning [23]. A simple way is to compare the results of each learner on the whole evaluation dataset. In this study, there are two target variables for prediction and the diversity for a learner *h* given that a dataset *D* is defined as follows:

(4) $$\begin{array}{c} {Div}_{h}=\frac{\sum_{i=1}^{\left|D\right|}{\left(h\left(x_{i}\right)-h_{ens}\left(x_{i}\right)\right)}^{2}\cdot \phi \left(h\left(x_{i}\right),\left(y_{i},z_{i}\right)\right)}{{\left|D\right|}^{2}}; \end{array}$$

since *h*(*x*_*i*_) returns a tuple, in the definition we use Hamming distance when evaluating the difference between two tuples. *ϕ*(·, ·) is an indicator function in which *ϕ*(*h*(*x*_*i*_), *y*_*i*_) = 1 if *h*(*x*_*i*_) = *y*_*i*_, and 0 otherwise. *h*_ens_ stands for the ensemble learner of majority voting. The intuition of this definition is that if the output of a learner *h* is away from that of the ensemble learner *h*_ens_, it is assigned with large diversity [24].

To this end, we are able to sort all learners by both their accuracy and diversity. We use a sorting strategy named nondominated sort (NDS) to get a reasonable sorting. The rule NDS is that if the accuracy and diversity of *h*_*i*_ can dominate those of *h*_*j*_, *h*_*i*_ should be ahead of *h*_*j*_ in the queue. When the accuracy and diversity of *h*_*i*_ and *h*_*j*_ cannot dominate each other, we add the rank of accuracy and diversity to form a single rank *r*. And the learner of small *r* should be ahead of the other [25].  Table 1 shows an example of 6 learners sorted by NDS.

In Table 1, the column Sum Rank stands for the sum of rank of accuracy and diversity of an individual learner. And the column NDS Rank stands for the ranking by NDS algorithm. Learner 1 dominates Learner 2 at both the rankings of accuracy and diversity. Hence the ranking of Learner 1 is prior to Learner 2. But Learner 3 and Learner 4 cannot dominate each other. In such case, NDS uses the Sum Rank for sorting, which adds the ranking of accuracy and diversity together. Finally, we get a fully sorted list of all learners in the base set, and we select the top *b*% of the base set size to form an ensemble learner.

### 2.3. Deep Boosting

With the definition of accuracy and diversity of the base learners, we can sort the learners based on their quality. To further get an optimal weight for combination, an iterative procedure can be applied to search valuable data samples in the training dataset as well as updating the weights. Adaboost is a famous algorithm to find optimal ensemble weights.  Algorithm 1 shows the main steps of Adaboost.

In Adaboost, a uniform distribution *V* is imposed on the training dataset *D*. Each round the combination weights *α* and the distribution *V* are both updated according to the performance of the current learner on the whole training dataset. If a sample is misclassified by some learners, it would be chosen again with high probability, which is controlled by the distribution *V*.

When it comes to deep ensemble learning, a different sample selection and weight update strategy is implemented. The main idea of deep ensemble learning is described as follows. Firstly the initial distribution *V* is set to *V*_*i*_ = 1/|*D*|. Then try to solve the optimization problem as follows:

(5) minα≥0 1nΦ1−yj∑i=1nαihixj+λ∑i=1nαiris.t. ∑i=1nαi≤1n.

Cortes et al. [26] proposed an algorithm to solve the above optimization problem, and a vector of optimal weights can be determined. Finally, for a test example *x*′, the result can be *y*′ = (1/*n*)∑_*i*=1_^*m*^ *h*_*i*_(*x*′). For a binary output, a sign function *s* can be applied on *y*′, in which *s*(*y*′) = 1 if *y*′ ≥ 0.5 and 0 otherwise.

### 2.4. Base Learners

The quality of base learners affects the performance of the ensemble significantly. In this study, we use two kinds of base learners. The first is decision tree (DT) and the second is support vector machine (SVM). Note that both types of learners implement shallow models with two layers. For DT, a path from a leaf to the root is in fact a conjunctive normal form (CNF), and the root performs an OR operation of all paths in the tree; that is, *h*_DT_ = ⋃_*i*=1_^*p*^ *c*_*i*_. For SVM, the model is structured with a kernel operation between the test sample *x*_*t*_ and the samples of the training dataset *D* and then summarizes with a normalized weight vector; that is, *h*_SVM_(*x*_*t*_) = ∑_*i*=1_^|*D*|^ *α*_*i*_ *k*(*x*_*t*_, *x*_*i*_). For either DT or SVM, a three-layer model can be obtained by ensemble the trained base learners with a vector of learned weights.

The DT and SVM models are implemented by the famous WEKA project [27]. And in order to be invoked in MATLAB environment, we use the Spider project to generate a MATLAB interface for WEKA. To train each learner, a sampling procedure is launched on the training dataset *D* with replacement, resulting in some difference between the training datasets of each learner. The size of the set of base learners is denoted as *m*, including *m*_DT_ DTs and *m*_SVM_ SVMs with default parameter settings. In our evaluation, we set *m*_DT_ = 500 and *m*_SVM_ = 500 to build a relative large set of base learners, leading to a sufficient ensemble.

## 3. Evaluations

### 3.1. Dataset and Settings

We evaluate the proposed on a real clinical dataset gathered from some veteran TCM doctors, composing 2835 records. There are 21 different types of diseases in the dataset attached with 4 kinds of feature groups. The first group is the ICD-10 label vector. There are 31 ICD-10 labels concerning this study. But for each data record, there is only one ICD-10 label that can be attached. We use a boolean vector with 31 elements to indicate which ICD-10 label is attached among all labels. The second group contains the patient's information, including age, gender, job type, history of disease, weight, and height. All this information is placed in a real vector with 11 elements. The third group contains the diagnosis and ZHENG description of the patient in Chinese. The raw data of this field is in plain text which is not easy to process directly. We process them with a key word matching procedure. 4000 key words including the name of diseases, name of acupoints, ZHENG description, and severity description are predefined. And the diagnosis description text is matched with the set of key words. A boolean vector records the matching result whose element indicates whether the corresponding word exists in the text description. Finally the fourth group describes the acupoints proposed by the doctor for acupuncture treatment. In this study 53 acupoints are considered for analysis.  Table 2 shows the feature of the evaluation dataset.

To make a clear presentation, Table 3 shows some examples of the dataset. Note that the name of acupoints and diagnosis description are originally in Chinese. We translate them into English for presentation in the table.

### 3.2. Evaluation Criteria and Methods for Comparison

To evaluate the effectiveness of the proposed method, we perform two types of evaluation. The first is to evaluate the prediction of ICD-10 labels given a diagnosis description and patient's basic information, as well as the acupoints for treatment. A zero-one loss function is adopted to evaluate the accuracy of the model output. Equation (6) shows the accuracy evaluated by a zero-one loss function:

(6) $$\begin{array}{c} \text{Acc}\left(\;h,D\;\right)=\frac{1}{n}\overset{n}{\underset{i=1}{\sum }}\delta \left(\;h\left(\;x_{i}\;\right),y_{i}\;\right), \end{array}$$

where *D* = {(*x*_1_, *y*_1_), (*x*_2_, *y*_2_),…, (*x*_*n*_, *y*_*n*_)}. *δ*(·, ·) is an indicator function where *δ*(*y*_*i*_, *y*_*j*_) = 1 if *y*_*i*_ = *y*_*j*_ and 0 otherwise.

For the second type of evaluation, we want to illustrate the effect of acupoint recommendation for a treatment plan given the basic information of a patient. This type of evaluation can be regarded as a multilabel classification problem. In this case, we adopt a Hamming loss to evaluate the accuracy. Equation (7) gives the definition of the Hamming loss:

(7) $$\begin{array}{c} {Loss}_{H}\left(\;h\left(\;x\;\right),y\;\right)=\frac{1}{\left|y\right|}\overset{\left|y\right|}{\underset{i=1}{\sum }}\left(\;h\left(\;x\;\right)\Delta y\;\right). \end{array}$$

In (7), *y* is the ground truth labels associated with *x*, and *h* is the learner to be evaluated. The Hamming loss function evaluates how many sample-label pairs are misclassified by the learner *h*.

We also implement two current state-of-the-art methods for the problem to be tackled in this paper and evaluate them on the same dataset, to further show the effectiveness of the proposed method. The first method is the multiview KNN method proposed by Liang et al. [9]. The second is a deep learning based method, which proposed a convolutional neural network for healthcare data decision making [28]. The motivation of choosing these two methods is twofold. The first is that both of them (Liang et al. [9, 28]) are proposed for TCM data analysis, which is similar to the theme of this study. And the evaluation dataset is the same as that used in this study. The second is that these two methods reflect two different directions for medical data analysis. The multiview KNN method in fact obeys the local learning and ensemble learning principles, leading to shallow model and transductive learning, which means that it is not necessary to derive a general model for the problem. The convolutional neural network method attempts to derive a classification function of powerful ability so as to express arbitrary complex classification boundary. For brevity, we denote these two methods as MV-KNN and CNN. The parameters of MV-KNN and CNN are set to default as they are proposed.

### 3.3. Evaluation Results

We use a tenfold validation strategy for evaluation. The whole dataset is randomly divided into 10 parts with equal sizes. In each round, 9 parts are used to train the model and the remainder for test. We randomly divide the dataset 20 times. For each time a tenfold validation is run. Totally there are 200 runs. The mean loss and stand derivation are recorded in either kind of evaluation.  Table 4 shows the ICD-10 annotation accuracy of each type of disease.

The column DEL stands for the accuracy of the proposed method. In Table 4, we boldface the best result in each row. At the last of the table, we summarize the accuracy of three methods. It can be seen that the proposed method has best performance in the annotation of 17 (totally 21) types of diseases. Moreover, in a multiple-label classification perspective, the proposed method also achieves the best result for all diseases to be annotated, as shown in the last row of Table 4. It can be concluded that the proposed method is effective for the annotation of the concerned diseases. The proposed method achieves best performance among all three methods for 17/21 ≈ 81.0% types of disease and for average results of all diseases, which indicates that the proposed method is statistically better than the other two methods.

For the second part of evaluation, we want to see the accuracy of acupoints recommendation for treatment. We compare the ground truth acupoints suggested by experienced doctors with the model output. Note that in this part MV-KNN and CNN are not suitable for this case. Henceforth we only report the accuracy measured by Hamming loss and the variance of the whole accuracy of the proposed method.  Table 5 shows the results of this session of evaluation.

## 4. Conclusions

In this paper, we proposed an ensemble learning framework for ICD-10 label annotation and acupoints recommendation. The model analyzes the clinical diagnosis records in plain text, acupoints for acupuncture treatment, and the patient's basic information and performs multilabel classification to annotate correct ICD-10 labels for each clinical record. At the same time, the model recommends acupoints for personal treatment, which provides valuable support for doctor's diagnosis decision. The proposed method adopts the recently proposed deep ensemble learning to find the optimal weight vector for combination of base learners. Different from the traditional Adaboost method, the deep ensemble learning can achieve better generalization ability when given a set of base learners with powerful representation ability. Decision tree and support vector machine classifiers are implemented as the base learners. We set up our evaluation on a real clinical dataset gathered from several veteran doctors, with comparison to two previously proposed successful methods. We achieve an accuracy of 88.2% in ICD-10 labels annotation evaluated by the zero-one loss function and 79.6% in acupoints recommendation evaluated by the Hamming loss function, either of which is superior to the two previous methods.

## Figures and Tables

**Algorithm 1:**
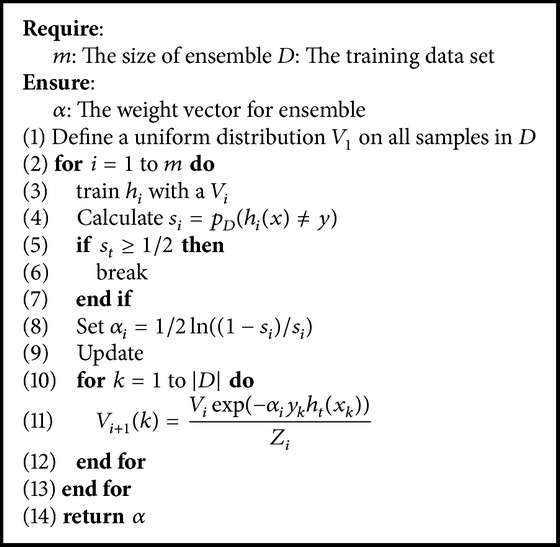
Adaboost.

**Table 1:** An example of 6 learners and their rankings.

Learner number	Accuracy rank	Diversity rank	Sum rank	NDS rank
Learner 1	1	2	3	1
Learner 2	2	3	5	3
Learner 3	5	4	9	4
Learner 4	4	6	10	5
Learner 5	3	1	4	2
Learner 6	6	5	11	6

**Table 2:** Description of the evaluation dataset.

Number	Name	Type	Description
1	ICD-10 label	Boolean vector	The ICD-10 labels associated with the record
2	BasicInfo	Real vector	Patient's basic information, 11-ary
3	Diagnosis text feature	Boolean vector	4000-ary
4	Acupoints	Boolean vector	Acupoints in the patient's acupuncture plan, 53-ary

**Table 3:** Description of the evaluation dataset.

No.	Name	Sample data
1	ICD-10 labels	Fibromyalgia: M79.7
2	BasicInfo	Age: 33, gender: male, weight: 68, height: 171, and job type: heavy
3	Diagnosis text	The sequela of stroke hemiplegia: there has been some recovery, for many years has not double knee joint pain, were migratory, Jigzhi healed, recently accompanied by low back pain, pale tongue slightly red, and moss white veins fine strings
4	Acupoints	Huantiao, Yinmen, Taixi, Yaoyangguan, Changqiang, and YangChi (right)

**Table 4:** ICD-10 annotation accuracy of each type of disease (%).

No.	Name	Size	DEL	MV-KNN	CNN
1	Arthralgia syndrome	481	82.4 ± 2.7	83.1 ± 2.8	**84**.**2** ± **3**.**4**
2	Acne	75	**90**.**2** ± **2**.**1**	86.4 ± 2.3	85.3 ± 3.1
3	Epilepsy	26	**89**.**1** ± **2**.**5**	88.0 ± 1.9	86.9 ± 2.4
4	Tinnitus and deafness	68	83.1 ± 2.6	81.0 ± 3.1	**85**.**2** ± **3**.**6**
5	Abdominal pain	96	**84**.**7** ± **2**.**7**	81.3 ± 2.9	82.8 ± 3.4
6	Allergic rhinitis	376	**89**.**2** ± **2**.**1**	84.1 ± 2.8	85.3 ± 3.0
7	Neck and shoulder pain	110	**91**.**4** ± **1**.**9**	88.4 ± 2.1	86.0 ± 2.5
8	Cervical spondylosis	33	**92**.**6** ± **2**.**2**	87.7 ± 2.9	90.5 ± 3.1
9	Cough	96	**88**.**5** ± **2**.**7**	86.9 ± 3.1	87.1 ± 3.9
10	Facial paralysis	89	**82**.**7** ± **1**.**3**	78.8 ± 2.5	79.1 ± 3.0
11	Traumatic brain injury	47	85.8 ± 2.1	**86**.**0** ± **2**.**9**	85.1 ± 2.6
12	Migraine	33	**93**.**0** ± **2**.**9**	88.7 ± 3.2	89.4 ± 3.6
13	Ankylosing spondylitis	33	**91**.**9** ± **2**.**0**	90.0 ± 3.6	91.1 ± 3.9
14	Insomnia	47	**90**.**2** ± **2**.**2**	84.5 ± 3.3	88.5 ± 3.6
15	Headache	145	86.6 ± 2.5	87.1 ± 3.2	**89**.**2** ± **3**.**8**
16	Flaccidity syndrome	124	**87**.**2** ± **1**.**9**	83.1 ± 2.8	84.4 ± 3.1
17	Stomachache	145	**89**.**2** ± **2**.**4**	86.5 ± 2.8	87.2 ± 3.1
18	Asthma	355	**90**.**6** ± **2**.**1**	88.2 ± 2.9	88.6 ± 2.9
19	Palpitation	33	**90**.**2** ± **2**.**5**	89.9 ± 3.1	86.5 ± 3.4
20	Lumbocrural pain	397	**88**.**1** ± **2**.**3**	82.1 ± 3.2	87.2 ± 3.8
21	Urticaria and rubella	26	**85**.**4** ± **2**.**3**	84.2 ± 3.1	84.9 ± 3.0

22	Total	**2835**	**88**.**2** ± **2**.**8**	85.6 ± 3.4	86.4 ± 3.9

**Table 5:** Acupoints recommendation accuracy of each type of disease (%).

No.	Name	Accuracy	No.	Name	Accuracy
1	Arthralgia syndrome	77.9	2	Acne	82.3
3	Epilepsy	80.6	4	Tinnitus and deafness	75.6
5	Abdominal pain	76.8	6	Allergic rhinitis	77.2
7	Neck and shoulder pain	81.0	8	Cervical spondylosis	80.5
9	Cough	82.4	10	Facial paralysis	76.5
11	Traumatic brain injury	79.1	12	Migraine	78.4
13	Ankylosing spondylitis	78.3	14	Insomnia	81.4
15	Headache	80.0	16	Flaccidity syndrome	75.9
17	Stomachache	83.0	18	Asthma	81.2
19	Palpitation	82.2	20	Lumbocrural pain	80.1
21	Urticaria and rubella	77.9	—	—	—

22	Total	**79.6** ± **3.6**	—	—	—
